# A personal perspective of patient-centred clinical trials

**DOI:** 10.18632/oncotarget.28776

**Published:** 2025-11-14

**Authors:** Trevor Tyne, Elizabeth Ivimey, Leanne Duggan, Jia Liu

**Affiliations:** ^1^The Kinghorn Cancer Centre, St Vincent’s Hospital, Sydney, NSW, Australia; ^2^School of Clinical Medicine, Faculty of Medicine and Health, University of New South Wales, Sydney, Australia; ^3^Garvan Institute of Medical Research, Sydney, NSW, Australia; ^*^These authors contributed equally to this work

**Keywords:** precision oncology, patient-centric care, clinical trials, tumor agnostic, drug development

## Abstract

Key objective: To illustrate the first-hand journey of three early phase trial participants highlighting their benefits and challenges of participation and patient-centric innovations required to improve trial experience.

Knowledge generated: Early phase trials have traditionally centred on dose-finding and toxicity. However as they have increased in number and improved in therapeutic intent, the patient experience becomes increasingly important. This article illustrates benefits of participation including access to novel therapies, support and close monitoring but challenges around eligibility criteria, finances, and communication. Proposed solutions including trial navigators, enhanced communication training, and greater flexibility in enrolment criteria to improve trials access.

## INTRODUCTION

Early phase clinical trials are crucial for advancing cancer therapeutics, yet the patient experience in these trials remains understudied. Traditionally EPCTs were reserved for patients with refractory/rare cancers where standard-of-care therapies are ineffective or no longer exist. However in more recent years, EPCTs are increasingly moving forwards in the treatment sequence for many settings particularly due to improvements in biomarker-matched therapies [1, 2], and initiatives such as FDA’s Project FrontRunner [3]. As such, understanding and prioritising the patient experience and perspective in biomarker-matched trials are critical to ensure these trials are conducted with the patient is at the centre of the study design is critical [4]. Tumor-agnostic trials provide a unique opportunity for patients with rare cancers harbouring biomarkers that inform therapeutic opportunities, to access genomically matched therapies. Although tumor-agonistic trials still represent a small number of EPCT opportunities and drug approvals, these approaches, often underpinned by biomarker-selection and molecular stratification, often result in higher response rates >20% and more meaningful clinical benefit [5]. Here, we provide a first-hand account of the perspectives of three individuals who have participated in biomarker matched trials. Our journeys through these trials reveals both their promise and opportunities for improvement in trial conduct to ensure these are patient-centric.

### Trevor’s story

My journey into the realm of early-phase clinical trials has been marked by unexpected challenges and profound hope. Diagnosed with leiomyosarcoma, a rare and aggressive cancer, I faced significant hurdles due to its unpredictable nature. The initial years were fraught with misdiagnoses and a lack of clarity regarding available treatment options. Like many patients with rare cancers, accessing advanced treatments was challenging, as standard care options were either ineffective or posed risks incompatible with my pre-existing medical conditions.

A pivotal moment occurred when I was introduced to an early phase clinical trials unit where I had the opportunity to enrol in a Phase 1 clinical trial for an investigational immunotherapy drug combination. This choice was made due to the absence of molecular biomarkers in genomic sequencing of my tumor, and despite the tumor being microsatellite stable and TMB-low, there are known benefits of immunotherapy in some soft tissue sarcomas [6]. This trial offered avenues beyond conventional chemotherapy, which was not viable due to my cardiac conditions, and the fact that immunotherapy is not reimbursed in my country for my cancer type. The decision to participate was daunting, involving uncertainty, rigorous screenings, and a demanding schedule of monitoring and assessments. However, it also provided access to cutting-edge research, exceptional medical supervision, and a chance to contribute to future advancements in cancer treatment.

### Beth’s story

I was diagnosed in late December 2022 with intrahepatic cholangiocarcinoma after being investigated for shortness of breath and cough. This diagnosis was confirmed by biopsy and I commenced routine chemotherapy & immunotherapy in January 2023. I was offered the opportunity to enter a national genomic tumour testing program, and a FGFR2-fusion in my tumour was identified. I completed 8 cycles of chemotherapy/immunotherapy and had stable disease – no tumour regression. I entered a tumor-agnostic clinical trial that was able to successfully target the fusion, and on first imaging after 8 weeks the tumour had reduced in size by 31%. As a participant of the clinical trial, I was offered every opportunity for inclusion, time, explanations, solutions to side effects and unending support. Despite extensive side effects I was supported with a comprehensive multidisciplinary toxicity management team, which went above and beyond to troubleshoot side effects so that I could receive the dose intensity required for tumour control.

At six months into the trial I had a new single metastasis and was successfully treated with targeted radiotherapy so that I could continue drug therapy. I showed progression with new disease at 12 months after a break due to side effects, and I am fortunate to now receive a new pan-FGFR inhibitor on compassionate access which is working well.

I will do anything within my ability to encourage people to have genomic testing and to get the right treatment for their cancer, especially participation in clinical trials. Contributing to medical research gave us a sense of purpose and satisfaction helping future patients. Despite published data on time toxicity [5], I personally do not feel that I was ever wasting my time in healthcare settings; I was provided with additional support and security. I do enjoy a great quality of life with family and friends; have travelled to Europe, had multiple holidays within Australia; all of this has been possible thanks to being a participant in a clinical trial.

### Leanne’s story

I am a full-time senior case worker at the NSW domestic violence crisis line and a strong advocate - both for myself and others - about equity of access in the health system and other areas. Born with congenital defects and blindness, I have navigated a life overcoming adversity, but none has been greater than my diagnosis with advanced pancreatic cancer. Despite extensive surgery and chemotherapy, my cancer progressed and last year, I was running out of time when 2nd line standard chemotherapy was running out of steam. Self-funded molecular testing I had arranged and paid for, identified a KRAS G12D mutation, and I was fortunate enough to meet a cancer trials team that had a tumor-agnostic clinical trial targeting this mutation across different cancer types.

However, the trials team had to overcome significant hurdles to get me enrolled as a participant and overcome barriers that many people living with a disability face. Mandated trial biopsies had to be done under a general anaesthetic with a specialist anaesthetist due to needle phobia arising from extensive medical interventions as a child. As the trial medication is a tablet, I have used my braille labeller and computer to identify medications and record my dosage and pill diary. The trials team received specific training on supporting the care needs of visually impaired patients and making myself and my guide dog Ester feel safe, via the support of Guide Dogs NSW/ACT. Fortunately, the clinical trial has successfully eliminated cancer pain I was experiencing prior to enrolment, and my tumor marker has reduced 100-fold, and cancer shrunk significantly on recent scans. Despite the demands of being a trial participant I have been able to continue to work full time and enjoy a fruitful social life with my extensive support network of friends.

### The quintessential value of foundational ethics and the role of core values

Over many years it became increasingly evident that clinical intuitions need to have a strong ethical foundation, based on values external to those created by that institution. Clinical trials represent a complex interplay of medical ethics, patient care, and scientific innovation. At the heart of ethical medical practice lies an overarching value system that guides decision-making and patient interactions. Institutions grounded in strong ethical frameworks, such as compassion, justice and integrity often prioritise compassion and holistic care. In contrast, those lacking such foundational ethics may adopt a more process-driven approach, potentially overlooking the individual needs of patients.

The quintessential importance of such a value system cannot be overstated, as it fosters an environment where patients feel genuinely cared for, beyond mere clinical protocols.

### Benefits of participation in early-phase clinical trials

1. *Comprehensive monitoring and high-quality care*: One of the most profound aspects of our clinical trial experience was the exceptional level of care and support provided by the medical team. The oncologists, trial coordinators, and nursing staff displayed unparalleled compassion and professionalism. Unlike traditional oncology settings, where visits may be brief, the trial environment allowed for in-depth discussions, proactive monitoring, and meticulous management of side effects. This approach enhanced our sense of safety and deepened our trust in the process.
2. *Access to novel therapies and expanded treatment options*: With limited standard treatment options, participating in tumor-agnostic clinical trials and receiving targeted therapy opened new possibilities for us. Even when disease progression occurred, the trial’s structure allowed for continuous adjustments to treatment plans fostering optimism. In contrast to conventional perceptions regarding strict trial protocols, in the early phase setting, this flexibility and negotiation between the trials team and the sponsor, contrasted sharply with conventional treatment pathways, where options might be exhausted more quickly.
3. *Psychological and emotional benefits of trial participation*: Engaging in a clinical trial provided a sense of empowerment, knowing we were actively contributing to advancing cancer research and helping those patients following us. The structured care approach and the presence of a dedicated team alleviated feelings of isolation often experienced by cancer patients. Contrary to the perception that clinical trials are impersonal and time-toxic [7], our experience was profoundly person-centred and additional time interacting with the healthcare system engineered a sense of security and support. However we note that our experience is coloured by ‘survivor bias’ as we have all been on trials for >6 months. In contrast, median participation on EPCTs per patient is around 2.5 months, with 7–10 days dedicated to research/monitoring/assessment days required for the trial [8], and as such these time burdens can be considerable especially for those who do not benefit from EPCTs.

### Challenges and recommendations for improvement

While the benefits of our clinical trial experience were significant, several challenges need addressing to make early-phase trials more patient-friendly and accessible:

1. *Barriers to eligibility and access*: Many patients are excluded from trials due to stringent eligibility criteria, even when they might benefit from the investigational treatment or harbour a biomarker matching a tumor-agnostic therapy. Not all clinical trials include a tumor-agnostic cohort, due to financial and market incentives to demonstrate efficacy in a more common cancer type, that can be more profitable for the company. A more flexible and personalised approach to patient selection, embracing tumor-agnostic efficacy, and considering real-world complexities, could expand access to potentially life-saving therapies.
  A major barrier to access is the lack of awareness about clinical trials. Collaboration with diverse stakeholders can foster innovation, improve care coordination, and enhance the patient experience and toxicity management. As the landscape of oncology trials providing ground-breaking therapies that can enhance or complement what is available as ‘standard’ or ‘funded’ care in many jurisdictions is changing, we advocate for patient-facing material that is translated to many languages, including videos showcasing patient stories for patients/families to refer to help them understand the merits and challenges of participating on a clinical trial.
  In addition to the oncology team, family physicians play a critical role in referring patients to specialist care and supporting them throughout their cancer journey. We recommend resources to support non-cancer healthcare professionals on education and resources regarding the changing landscape of cancer therapy, services, clinical trials and support options and ensure that medications/procedures initiated do not contradict or interfere with trial treatments.
2. *Financial and logistical burdens*: The financial implications of trial participation—travel expenses, time off work, and accommodation costs for those distant from trial sites—are often underestimated. Establishing financial support programmes, telehealth options for routine monitoring, and logistical assistance could significantly reduce these barriers. Reimbursement for out-of-pocket costs associated with trial participation including medications, consultations to manage side effects, travel and accommodation is well-supported by sponsors of most pharmaceutically-run trials, but are less available for investigator-led, academic-run trials which often function on more limited budgets and this should be matched to ensure that patients are not discouraged to participate in the meaningful research that are run by academic research organisations.
3. *The role of clinical trial navigators*: For many patients, the clinical trial process is complex and overwhelming. A dedicated trial navigator—a trained professional assisting patients in understanding protocols, managing appointments, and coordinating care—could streamline the experience. Navigators could also facilitate transitions between trials, preventing gaps in treatment.
  Navigators play an educational role to facilitate awareness and improve communication skills on the merits and challenges of clinical trials. Navigation is particularly important for underserved populations such as culturally/linguistically diverse populations and those from regional/rural oncology units where awareness about the benefits and access of trials is limited, and additional logistical hurdles e.g. interpreters, transport and accommodation need to be arranged.
  Studies have shown that cancer navigation programmes can improve patient outcomes, reduce anxiety and depression, and enhance patient satisfaction [9]. Trial navigators can help to onboard patients and reduce fear and anxiety in new patients by providing personalised support and guidance.
4. Enhancing communication and transparency: While we have been fortunate to have a highly communicative medical team, many trial patients report a lack of transparency regarding test results, treatment expectations, and long-term follow-up. Ensuring clear, patient-friendly communication and real-time updates on trial progress could enhance the overall patient experience. Patients deserve to be active participants in their treatment journey, rather than passive subjects in research.
  Communication skills training should be a top priority for new trials staff entering a research unit as it is very difficult to speak openly with cancer patients without experience. Empathy is essential in healthcare, as it can improve patient outcomes, satisfaction, trust and adherence to treatment recommendations. Given the high turnover of staff working in the clinical trials setting, and not all trials staff having healthcare backgrounds that are patient-facing, we recommend that trial units provide regular training on effective communication, active listening, and emotional intelligence. Complex discussions regarding risk and uncertainty of trial treatment and toxicity, the implications of slots and wait time for enrolment, randomisation and informed consent are important. This should be a competency task during induction, and yearly updates for all staff as part of annual reviews.

### Final reflections: The future of patient-centred trials

Clinical trials are often perceived as a last resort, but they should be considered earlier in the treatment sequence, particularly as personalised medicine continues to evolve. Our experiences underscore the need for patient-centred trial designs, where participants’ perspectives and needs inform the development and execution of clinical studies. The importance of patient-centred, continuity of care across the trial journey, and adequate handover of patients especially when moving between trial centres and treating teams when commencing or completing clinical trial treatment cannot be understated. It is reassuring to see the increasing use of patient-reported outcomes in toxicity assessment in early phase trials [10] and this should increase as EPCT therapies are moved forwards in the treatment paradigm for cancer patients.

While no trial guarantees success, the level of support, access to innovative therapies, and sense of contribution to medical progress can be profoundly meaningful. Moving forward, the clinical trial landscape must prioritise accessibility, patient empowerment, and compassionate care. While EPCTs remain drug-centered to define if a compound is active and safe, there is a need to share the objective of trials to being more patient-centred, with a review of protocol designs by patient advocates and PROs incorporated as endpoints. By embracing transparency, inclusivity, and patient advocacy, we can transform clinical trials into a true partnership between medical research and the individuals it seeks to serve.

## References

[1] Global Trends in R&D 2024: Activity, Productivity, and Enablers. February 2024. IQVIA Institute for Human Data Science. 2024.

[2] Subbiah V . The next generation of evidence-based medicine. Nat Med. 2023; 29:49–58. 10.1038/s41591-022-02160-z. 36646803

[3] Moon H , Zang DY , Ryu MH , Seo Y , Oh B , Hwang S , Farrand L . FDA regulatory considerations for oncology drug development. Pharmacol Res Perspect. 2024; 12:e1254. 10.1002/prp2.1254. 39075638 PMC11286537

[4] Sawyer C , Preston L , Taylor S , Davies M , Carter L , Krebs M , Cook N , Graham D , Thistlewaite F , Yorke J . Oncology patients’ experiences in experimental medicine cancer trials: a qualitative study. BMJ Open. 2021; 11:e047813. 10.1136/bmjopen-2020-047813. 34610932 PMC8493921

[5] Westphalen CB , Martins-Branco D , Beal JR , Cardone C , Coleman N , Schram AM , Halabi S , Michiels S , Yap C , André F , Bibeau F , Curigliano G , Garralda E , et al. The ESMO Tumour-Agnostic Classifier and Screener (ETAC-S): a tool for assessing tumour-agnostic potential of molecularly guided therapies and for steering drug development. Ann Oncol. 2024; 35:936–53. 10.1016/j.annonc.2024.07.730. 39187421

[6] Saerens M , Brusselaers N , Rottey S , Decruyenaere A , Creytens D , Lapeire L . Immune checkpoint inhibitors in treatment of soft-tissue sarcoma: A systematic review and meta-analysis. Eur J Cancer. 2021; 152:165–82. 10.1016/j.ejca.2021.04.034. 34107450

[7] Nindra U , Shivasabesan G , Childs S , Yoon R , Haider S , Hong M , Cooper A , Roohullah A , Wilkinson K , Pal A , Chua W . Time toxicity associated with early phase clinical trial participation. ESMO Open. 2023; 8:102046. 10.1016/j.esmoop.2023.102046. 37979324 PMC10774969

[8] Iskander R , Magnan Robart A , Moyer H , Nipp R , Gupta A , Kimmelman J . Time Burdens for Participants With Advanced Cancer in Phase I Trials: A Cross-Sectional Study. JCO Oncol Pract. 2025; 21:391–99. 10.1200/OP.24.00334. 39353144

[9] Wells KJ , Campbell K , Kumar A , Clark T , Jean-Pierre P . Effects of patient navigation on satisfaction with cancer care: a systematic review and meta-analysis. Support Care Cancer. 2018; 26:1369–82. 10.1007/s00520-018-4108-2. 29497815 PMC5898973

[10] Retzer A , Aiyegbusi OL , Rowe A , Newsome PN , Douglas-Pugh J , Khan S , Mittal S , Wilson R , O’Connor D , Campbell L , Mitchell SA , Calvert M . The value of patient-reported outcomes in early-phase clinical trials. Nat Med. 2022; 28:18–20. 10.1038/s41591-021-01648-4. 35039659

